# 10-Year Risk for Cardiovascular Disease Associated with COMISA (Co-Morbid Insomnia and Sleep Apnea) in Hypertensive Subjects

**DOI:** 10.3390/life13061379

**Published:** 2023-06-13

**Authors:** Laura Draelants, Camille Point, Benjamin Wacquier, Jean-Pol Lanquart, Gwenolé Loas, Matthieu Hein

**Affiliations:** Hôpital Universitaire de Bruxelles, Service de Psychiatrie et Laboratoire du Sommeil, Université Libre de Bruxelles (ULB), 1070 Bruxelles, Belgium; laura.draelants@ulb.be (L.D.); camille.point@hubruxelles.be (C.P.); benjamin.wacquier@hubruxelles.be (B.W.); secmed.psy.erasme@hubruxelles.be (J.-P.L.); gwenole.loas@hubruxelles.be (G.L.)

**Keywords:** cardiovascular risk, COMISA, insomnia disorder, obstructive sleep apnea syndrome, hypertension, polysomnography

## Abstract

Due to the few studies available, this study aimed to investigate the 10-year risk for cardiovascular disease (CVD) associated with COMISA (co-morbid insomnia and sleep apnea) in hypertensive subjects. Clinical data of 1009 hypertensive subjects extracted from the Sleep Laboratory database were analyzed. Framingham Risk Score ≥ 10% was used as a cut-off to identify hypertensive subjects with high 10-year risk for CVD. The association between 10-year risk for CVD and COMISA was investigated using logistic regression analyses. 65.3% of hypertensive subjects from our sample presented a high 10-year risk for CVD. After controlling for major confounding factors, multivariate logistic regression analyses demonstrated that unlike its components present separately, COMISA was significantly associated with high 10-year risk for CVD in hypertensive subjects (OR 1.88, 95% CI 1.01–3.51). In this study, we have demonstrated that the negative synergy between obstructive sleep apnea syndrome and insomnia disorder seems to play a central role in the 10-year risk for CVD in hypertensive subjects, which seems to indicate that the establishment of a systematic research and an adapted treatment of COMISA could open new perspectives to promote a better cardiovascular outcome in this specific subgroup of patients.

## 1. Introduction

Given the data currently available, there seems to be a bidirectional relationship between hypertension and obstructive sleep apnea syndrome (OSAS). Indeed, OSAS is a frequent comorbidity in hypertensive subjects and apneic subjects have a high prevalence of hypertension [1,2]. In addition, OSAS is a risk factor for hypertension whereas hypertension appears to predispose to the development of OSAS [3,4]. Some pathophysiological elements could explain this bidirectional relationship: (1) fluctuations of blood pressure in hypertensive individuals may promote obstructive respiratory events by a negative impact on the muscle tone of the upper airways, and (2) sleep fragmentation and intermittent hypoxia associated with OSAS may induce deleterious biological alterations (hyperactivation of the sympathetic nervous system, deregulation of the renin–angiotensin system, endothelial dysfunction, activation of inflammatory mechanisms and metabolic dysregulation) that play a central role in the pathophysiology of hypertension [5,6]. Moreover, in hypertensive subjects, the presence of comorbid OSAS seems to promote the occurrence of hypertensive crises, resistance to antihypertensive medication and cardiovascular events (fatal and non-fatal) [7,8,9]. However, although the presence of OSAS seems to be associated with a less favorable cardiovascular outcome in hypertensive subjects, it has been shown that the OSAS treatments had a limited impact on blood pressure control and cardiovascular prevention in this specific subgroup of patients [10,11]. Following these different elements, it therefore seems essential to carry out additional studies to allow a better identification of the potential cofactors involved in this higher cardiovascular risk associated with OSAS in hypertensive subjects.

In the literature, there is evidence for a negative synergistic effect between OSAS and insomnia disorder on the risk of cardiovascular disease (CVD) [12,13]. Indeed, compared to subjects with insomnia disorder or OSAS alone, subjects with COMISA (co-morbid insomnia and sleep apnea) appear to have a higher risk of CVD [14,15]. However, despite the frequent occurrence of COMISA in hypertensive subjects [16], no study has investigated the potential impact of this negative synergistic effect between OSAS and insomnia disorder on the risk of CVD in this specific subgroup of patients. Indeed, currently available studies have been limited to separately investigating the impact of OSAS and insomnia disorder on the risk of CVD in hypertensive subjects [17,18]. In this context, it could be interesting to study the impact of COMISA on the 10-year risk for CVD in hypertensive subjects to investigate the potential role played by this negative synergy between OSAS and insomnia disorder on cardiovascular outcome in this specific subgroup of patients.

This study aimed to investigate the 10-year risk for CVD associated with COMISA in hypertensive subjects to highlight a potential negative synergistic effect between OSAS and insomnia disorder on cardiovascular outcome in this specific subgroup of patients. Our hypothesis was that, compared to these components alone (insomnia disorder or OSAS), COMISA is associated with higher 10-year risk for CVD in hypertensive subjects. This approach was intended to provide reliable data to healthcare professionals regarding the potential role played by the negative synergy between OSAS and insomnia disorder on the 10-year risk for CVD in hypertensive subjects to promote a better cardiovascular outcome in this specific subgroup of patients.

## 2. Material and Method

### 2.1. Selection of Hypertensive Subjects

After application of inclusion and exclusion criteria selected for this study (Table 1) [19], 1009 hypertensive subjects who carried out a polysomnographic recording between 1 January 2022 and 31 December 2019 were extracted from the database of the Erasme Hospital Sleep Laboratory. Furthermore, we decided to extract only hypertensive subjects for this study since our objective was to focus on this specific subgroup of patients where comorbid sleep disorders may negatively impact cardiovascular outcome [17,18]. Finally, the recruitment procedure of hypertensive subjects referred to the Sleep Laboratory from outpatient sleep medicine consultations is described in Supplementary Data—Annex S1.

### 2.2. Medical and Psychiatric Assessment of Hypertensive Subjects

All hypertensive subjects benefited from a review of their medical records, a complete somatic assessment (clinical interview, physical examination and vital sign measurements) and usual complementary tests (blood test, urine analysis, electrocardiogram and electroencephalogram) to allow systematic screening of their potential comorbid somatic pathologies. Indeed, thanks to this complete somatic check-up, the traditional cardiovascular risk factors (Supplementary Data—Annex S2) have been systematically identified in all hypertensive subjects [20,21]. Furthermore, after repeated measurements of blood pressure (Supplementary Data—Annex S3) in all subjects on antihypertensive medication, the presence of mean systolic blood pressure <140 mmHg and mean diastolic blood pressure <90 mmHg was used to define controlled hypertension whereas the presence of mean systolic blood pressure ≥140 mmHg and/or mean diastolic blood pressure ≥90 mmHg was used to define uncontrolled hypertension [19].

Then, based on the clinical data collected during this complete somatic check-up, the 10-year risk for CVD was calculated using the Framingham Risk Score (FRS) in all hypertensive subjects [22]. Indeed, in the literature, there seem to be data in favor of this use of the FRS to assess cardiovascular risk in hypertensive subjects [23,24]. Thus, in hypertensive subjects, FRS < 10% was used to determine low 10-year risk for CVD and FRS ≥ 10% was used to determine high 10-year risk for CVD [22].

Moreover, in all hypertensive subjects, a systematic screening for their potential comorbid psychiatric disorders based on the diagnostic criteria of DSM-IV-TR (before 2013) and DSM 5 (after 2013) was carried out by a psychiatrist from our unit during a complete psychiatric interview [25,26].

Finally, all hypertensive subjects completed self-questionnaires to allow an assessment of their depressive symptoms, complaints of daytime sleepiness and symptoms of insomnia (Supplementary Data—Annex S4) [27,28,29].

### 2.3. Sleep Assessment of Hypertensive Subjects

After this comprehensive somatic and psychiatric assessment, an interview specifically focused on sleep habits and sleep-related complaints was conducted in all hypertensive subjects to systematically research for symptoms suggestive of the main sleep disorders.

Then, according to the stay conditions at the Sleep Laboratory, a polysomnographic recording meeting the criteria of the American Academy of Sleep Medicine was carried out in all hypertensive subjects (Supplementary Data—Annex S5) [30]. Finally, specialized technicians visually scored these polysomnographic recordings based on the criteria of the American Academy of Sleep Medicine (Supplementary Data—Annex S6) [31,32,33].

Thanks to this specific sleep interview and this polysomnographic recording, a systematic screening for potential comorbid sleep disorders was carried out systematically in all hypertensive subjects: insomnia disorder (Supplementary Data—Annex S7), OSAS (apnea-hypopnea index ≥ 5/h), COMISA, moderate to severe periodic limb movement syndrome (periodic limb movement index ≥ 15/h), restless legs syndrome and short sleep duration (<6 h) [34,35,36,37,38].

### 2.4. Statistical Analyses

Stata 14 was used for statistical analyses. Histograms, boxplots and quantile–quantile plots were used to check the normal distribution of the data whereas Levene’s test was used to check the equality of variances.

To allow our analyses, hypertensive subjects with FRS < 10% were included in the group with low 10-year risk for CVD whereas hypertensive subjects with FRS ≥ 10% were included in the group with high 10-year risk for CVD [22].

Continuous data were described by their median (P25–P75) and analyzed by non-parametric tests (Wilcoxon test) since most of these data were not distributed symmetrically. Categorical data were described by percentages and analyzed by Chi^2^ tests.

Univariate logistic regression models were used to investigate the association between 10-year risk for CVD and COMISA status as well as to determine potential confounding factors to be included in multivariate analyses (Supplementary Data—Annex S8). Thus, only significant confounding factors during univariate analyses were introduced hierarchically into the different multivariate logistic regression models to adjust the 10-year risk for CVD associated with COMISA status.

The Hosmer and Lemeshow test was used to check the adequacy of the final model whereas the Link test was used to check the specificity of the final model.

A *p*-value < 0.05 was used to identify significant results.

## 3. Results

### 3.1. Polysomnographic Data (Table 2)

Compared to hypertensive subjects with low 10-year risk for CVD, hypertensive subjects with high 10-year risk for CVD presented alterations in respiratory parameters (increase in oxygen desaturation index/apnea-hypopnea index/total time under 90% of SaO2), changes in sleep architecture (increase in stage 1 percentage/wake after sleep onset percentage/micro-arousal index and decrease in slow-wave sleep percentage/REM latency/sleep efficiency/total sleep time) and alterations in motor parameters (increase in periodic limb movement index). The other polysomnographic data showed no significant differences between the two groups of hypertensive subjects.

**Table 2:** Polysomnographic data (n = 1009).

	Whole Sample (n = 1009)	Group with Low 10-Year Risk for CVD (n = 350)	Group with High 10-Year Risk for CVD (n = 659)	*p*-Value
Sleep latency (min)	25.0 (14.0–49.0)	24.7 (14.7–49.0)	25.7 (13.7–48.5)	0.981
Sleep efficiency (%)	76.3 (66.2–83.2)	78.1 (69.9–84.5)	75.2 (64.0–82.6)	<0.001
Sleep period time (min)	450.0 (407.5–482.0)	450.0 (415.3–486.7)	449.5 (402.5–481.5)	0.109
Total sleep time (min)	375.5 (325.0–415.5)	384.0 (340.0–424.5)	370.0 (316.0–410.0)	<0.001
% stage 1	8.2 (5.3–11.5)	7.5 (5.0–10.2)	8.7 (5.5–12.0)	<0.001
% stage 2	54.9 (47.0–61.3)	55.3 (47.9–61.6)	54.2 (46.5–61.3)	0.223
% slow-wave sleep	1.7 (0.0–6.8)	3.3 (0.3–8.9)	1.1 (0.0–5.7)	<0.001
% REM sleep	15.2 (11.0–19.2)	16.0 (11.6–19.7)	15.0 (10.6–19.0)	0.051
REM latency (min)	84.3 (59.3–132.3)	88.3 (63.0–137.5)	81.5 (57.0–127.5)	0.025
% wake after sleep onset	14.8 (9.0–23.3)	12.8 (8.4–20.2)	15.6 (9.7–24.1)	<0.001
Number of awakenings	34 (23–49)	34 (23–48)	34 (23–50)	0.717
Micro-arousal index	13 (8–22)	11 (7–17)	15 (9–26)	<0.001
Apnea–hypopnea index	7 (2–20)	4 (1–10)	10 (3–25)	<0.001
Oxygen desaturation index	3 (1–9)	1 (0–4)	4 (1–12)	<0.001
Total time under 90% of SaO2 (min)	7.0 (0.5–47.7)	1.3 (0.0–18.0)	15.5 (1.5–72.7)	<0.001
PLMs index	2 (0–12)	2 (0–10)	3 (0–14)	0.040
	Median (P25–P75)	Median (P25–P75)	Median (P25–P75)	Wilcoxon Test

CVD = cardiovascular disease, PLMs = periodic limb movements during sleep, REM = rapid eye movement.

### 3.2. Demographic Data (Table 3)

In our sample, 65.3% of hypertensive subjects (n = 659) presented a high 10-year risk for CVD. Gender, body mass index categories, age, smoking, alcohol consumption, type 2 diabetes, dyslipidemia status, hypertension status, cardiovascular comorbidities, aspirin therapy, sleep movement disorders and COMISA status were significantly associated with high 10-year risk for CVD in hypertensive subjects. In addition, hypertensive subjects with high 10-year risk for CVD were characterized by higher age/body mass index/triglycerides levels and lower Insomnia Severity Index scores/HDL-C levels/Beck Depression Inventory scores than hypertensive subjects with low 10-year risk for CVD. The other demographic data showed no significant differences between the two groups of hypertensive subjects. Finally, in our sample, the rate of COMISA was high since the comorbid insomnia disorder was present in 38.4% of hypertensive subjects with OSAS.

**Table 3:** Sample description (n = 1009).

Variables	Categories	%	Group with Low 10-Year Risk for CVD	Group with High 10-Year Risk for CVD	*p*-Value Chi^2^	OR (CI 95%)	*p*-Value
Gender	Female (n = 339)	33.6%	60.6%	19.3%	<0.001	1	<0.001
	Male (n = 670)	66.4%	39.4%	80.7%		6.44 (4.82 to 8.59)	
Body mass index categories (kg/m^2^)	<25 (n = 173)	17.1%	24.9%	13.0%	<0.001	1	<0.001
	≥25 & <30 (n = 370)	36.7%	31.7%	39.3%		2.36 (1.63 to 3.42)	
	≥30 (n = 466)	46.2%	43.4%	47.7%		2.09 (1.46 to 2.98)	
Age (years)	<65 (n = 866)	85.8%	96.0%	80.4%	<0.001	1	<0.001
	≥65 (n = 143)	14.2%	4.0%	19.6%		5.84 (3.31 to 10.31)	
Smoking	No (n = 820)	81.3%	90.0%	76.6%	<0.001	1	<0.001
	Yes (n = 189)	18.7%	10.0%	23.4%		2.74 (1.85 to 4.07)	
Alcohol	No (n = 723)	71.7%	78.0%	68.3%	0.001	1	0.001
	Yes (n = 286)	28.3%	22.0%	31.7%		1.65 (1.22 to 2.23)	
COMISA status	No (n = 116)	11.5%	14.0%	10.2%	<0.001	1	<0.001
	Sleep deprivation alone (n = 67)	6.6%	6.3%	6.8%		1.50 (0.80 to 2.81)	
	Insomnia alone (n = 229)	22.7%	37.1%	15.0%		0.56 (0.35 to 0.88)	
	OSAS alone (n = 368)	36.5%	26.3%	41.9%		2.19 (1.42 to 3.40)	
	COMISA (n = 229)	22.7%	16.3%	26.1%		2.21 (1.37 to 3.55)	
Sleep movement disorders	No (n = 753)	74.6%	79.7%	71.9%	0.023	1	0.024
	Moderate to severe PLMs alone (n = 77)	7.6%	6.6%	8.2%		1.38 (0.83 to 2.30)	
	RLS alone or combined with PLMs (n = 179)	17.8%	13.7%	19.9%		1.61 (1.12 to 2.31)	
Excessive daytime sleepiness	No (n = 619)	61.3%	57.7%	63.3%	0.084	1	0.084
	Yes (n = 390)	38.7%	42.3%	36.7%		0.79 (0.61 to 1.03)	
Type 2 diabetes	No (n = 794)	78.7%	96.3%	69.4%	<0.001	1	<0.001
	Yes (n = 215)	21.3%	3.7%	30.6%		11.46 (6.43 to 20.43)	
Hypertension status	Controlled (n = 416)	41.2%	48.9%	37.2%	<0.001	1	<0.001
	Untreated (n = 365)	36.2%	42.3%	32.9%		1.02 (0.77 to 1.36)	
	uncontrolled (n = 228)	22.6%	8.8%	29.9%		4.44 (2.90 to 6.79)	
Dyslipidemia status	No (n = 382)	37.9%	51.7%	30.5%	<0.001	1	<0.001
	Without statin therapy (n = 348)	34.5%	28.3%	37.8%		2.26 (1.67 to 3.08)	
	With statin therapy (n = 279)	27.6%	20.0%	31.7%		2.69 (1.92 to 3.77)	
Cardiovascular comorbidities	No (n = 853)	84.5%	87.7%	82.9%	0.042	1	0.043
	Yes (n = 156)	15.5%	12.3%	17.1%		1.48 (1.01 to 2.16)	
Aspirin therapy	No (n = 802)	79.5%	88.0%	75.0%	<0.001	1	<0.001
	Yes (n = 207)	20.5%	12.0%	25.0%		2.45 (1.70 to 3.54)	
CRP (mg/L)	<1 (n = 235)	23.3%	25.7%	22.0%	0.184	1	0.185
	≥1 (n = 774)	76.7%	74.3%	78.0%		1.23 (0.91 to 1.66)	
Depression	No (n = 748)	74.1%	72.3%	75.1%	0.329	1	0.329
	Yes (n = 261)	25.9%	27.7%	24.9%		0.86 (0.64 to 1.16)	
Cardiovascular risk	Low (n = 350)	34.7%					
	Moderate to high (n = 659)	65.3%					
	**Median** **(P25–P75)**				**Wilcoxon test**		
BMI (kg/m^2^)	29.4 (26.2–33.3)		28.7 (25.0–32.9)	29.7 (26.8–33.8)	<0.001		
Age (years)	54 (47–60)		48 (45–55)	56 (51–62)	<0.001		
ESS	9 (5–13)		9 (5–13)	9 (6–13)	0.782		
BDI	4 (2–8)		4 (2–8)	3 (2–7)	0.018		
ISI	14 (9–18)		15 (10–19)	13 (9–17)	<0.001		
Cholesterol (mg/dL)	196 (172–220)		194 (169–218)	197 (173–220)	0.081		
HDL-C (mg/dL)	51 (43–62)		58 (48–71)	48 (40–56)	<0.001		
Triglycerides (mg/dL)	131 (94–187)		109 (80–156)	147 (105–200)	<0.001		
CRP (mg/L)	1.9 (1.0–3.7)		1.8 (1.0–4.0)	2.0 (1.0–3.6)	0.452		
Framingham Risk Score (%)	13.0 (8.0–21.0)		6.7 (5.0–8.2)	17.9 (13.1–25.6)	<0.001		

CVD = cardiovascular disease, OSAS = obstructive sleep apnea syndrome, COMISA = co-morbid insomnia and sleep apnea, CRP = C-Reactive Protein, PLMs = periodic limb movements during sleep, RLS = restless legs syndrome, ESS = Epworth sleepiness scale, BDI = Beck depression inventory, ISI = insomnia severity index.

### 3.3. Multivariate Regression Analyses (Table 4)

After hierarchical introduction of the different confounding factors identified during the univariate analyses, multivariate logistic regression analyses demonstrated that, unlike its components present separately, COMISA was significantly associated with high 10-year risk for CVD in hypertensive subjects.

**Table 4:** Multivariate analysis (n = 1009).

Variables	Model 1 OR Adjusted (CI 95%)	*p*-Value	Model 2 OR Adjusted (CI 95%)	*p*-Value	Model 3 OR Adjusted (CI 95%)	*p*-Value	Model 4 OR Adjusted (CI 95%)	*p*-Value
COMISA status		0.028		0.005		0.007		0.006
No	1		1		1		1	
Sleep deprivation alone	1.47 (0.71 to 3.06)		1.12 (0.48 to 2.59)		1.12 (0.48 to 2.61)		1.12 (0.48 to 2.61)	
Insomnia alone	0.92 (0.54 to 1.56)		0.74 (0.40 to 1.35)		0.73 (0.40 to 1.34)		0.73 (0.40 to 1.34)	
OSAS alone	1.50 (0.90 to 2.49)		1.61 (0.91 to 2.84)		1.57 (0.88 to 2.77)		1.58 (0.89 to 2.80)	
COMISA	1.85 (1.07 to 3.22)		1.91 (1.03 to 3.56)		1.87 (1.01 to 3.49)		1.88 (1.01 to 3.51)	

Model 1 = Model adjusted for gender, body mass index categories, age, smoking and alcohol. Model 2 = Model adjusted for gender, body mass index categories, age, smoking, alcohol, diabetes, hypertensions status, dyslipidemia status and cardiovascular comorbidities. Model 3 = Model adjusted for gender, body mass index categories, age, smoking, alcohol, diabetes, hypertensions status, dyslipidemia status, cardiovascular comorbidities and aspirin therapy. Model 4 = Model adjusted for gender, body mass index categories, age, smoking, alcohol, diabetes, hypertensions status, dyslipidemia status, cardiovascular comorbidities, aspirin therapy and sleep movement disorders. COMISA = co-morbid insomnia and sleep apnea, OSAS = obstructive sleep apnea syndrome.

### 3.4. Additional Multivariate Regression Analyses (Table 5)

After adjustment for the main confounding factors identified during the univariate analyses, additional multivariate logistic regression analyses revealed that, unlike the other categories studied, COMISA with short sleep duration was significantly associated with high 10-year risk for CVD in hypertensive subjects.

**Table 5:** Additional multivariate analyses (n = 1009).

Variables	%	Group with Low 10-Year Risk for CVD	Group with High 10-Year Risk for CVD	Model 1 OR Unadjusted (CI 95%)	*p*-Value	Model 2 OR Adjusted (CI 95%)	*p*-Value
COMISA status					<0.001		0.008
No	11.5% (n = 116)	14.0%	10.2%	1		1	
Short sleep duration alone	6.6% (n = 67)	6.3%	6.8%	1.50 (0.80 to 2.81)		1.12 (0.48 to 2.61)	
Insomnia without short sleep duration	13.8% (n = 139)	22.9%	9.0%	0.54 (0.33 to 0.89)		0.88 (0.46 to 1.71)	
Insomnia with short sleep duration	8.9% (n = 90)	14.3%	6.1%	0.59 (0.34 to 1.02)		0.52 (0.24 to 1.13)	
OSAS alone	36.5% (n = 368)	26.3%	41.9%	2.19 (1.42 to 3.40)		1.58 (0.89 to 2.80)	
COMISA without short sleep duration	12.1% (n = 122)	10.9%	12.7%	1.62 (0.95 to 2.75)		1.55 (0.76 to 3.15)	
COMISA with short sleep duration	10.6% (n = 107)	5.3%	13.3%	3.39 (1.83 to 6.28)		2.44 (1.10 to 5.42)	

Model 1 = Model unadjusted. Model 2 = Model adjusted for gender, body mass index categories, age, smoking, alcohol, diabetes, hypertensions status, dyslipidemia status, cardiovascular comorbidities, aspirin therapy and sleep movement disorders. COMISA = co-morbid insomnia and sleep apnea, OSAS = obstructive sleep apnea syndrome.

## 4. Discussion

Consistent with the available data [39,40,41,42], we have shown that the 10-year risk for CVD is high in hypertensive subjects. Indeed, in our sample, 65.3% of hypertensive subjects presented a high 10-year risk for CVD, which confirms the importance of this problem in this specific subgroup of patients compared to the general population [43]. However, this proportion of hypertensive subjects with high 10-year risk for CVD is lower than that of the study by Gómez-Marcos et al. (2009) (86.5%) [39], which could be explained by the inclusion in this study of hypertensive subjects at higher cardiovascular risk than in our study. Indeed, unlike our study, the hypertensive subjects included in the study by Gómez-Marcos et al. (2009) showed more frequent unhealthy lifestyle habits (smoking), higher occurrence of glycemic disorders (diabetes and glucose intolerance) and less use of antihypertensive/hypolipidemic medication [39]. Furthermore, the proportion of hypertensive subjects with high 10-year risk for CVD highlighted in our study is higher than that of the studies by de Paula et al. (2013) (26.0%) and Cesarino et al. (2012) (45.3%) [40,41], which could be explained by the inclusion in these two studies of hypertensive subjects at lower cardiovascular risk than in our study. Indeed, compared to our sample of hypertensive subjects, the samples used in the studies by de Paula et al. (2013) and Cesarino et al. (2012) to calculate the FRS had a higher proportion of women and did not include subjects with comorbid diabetes [40,41]. Finally, the proportion of hypertensive subjects with high 10-year risk for CVD demonstrated in our study is comparable to that of the study by Sato et al. (2014) (65.9%) which had included hypertensive subjects with a cardiovascular risk profile more comparable to that present in our sample of hypertensive subjects [42]. Thus, regardless of these differences in the recruitment of clinical samples, we confirmed that high 10-year risk for CVD was frequent in hypertensive subjects, which highlights the need to adequately identify the specific cardiovascular risk factors for this patient subgroup.

Similar to the literature, we have shown that the rate of insomnia disorder (45.4%) and OSAS (59.2%) was high in hypertensive subjects [44]. In addition, in hypertensive subjects with OSAS, we found that insomnia disorder was a frequent comorbidity (38.4%), which appears to be consistent with other studies currently available on COMISA [14,15]. Furthermore, similar to other specific subpopulations [15], we demonstrated that, unlike its components present separately, COMISA was associated with high 10-year risk for CVD in hypertensive subjects. However, some pathophysiological elements could help to better understand this frequent occurrence of COMISA and its negative impact on cardiovascular outcome in hypertensive subjects. First, one of the explanations for this high rate of COMISA demonstrated in our sample of hypertensive subjects could be the existence of some specific pathophysiological mechanisms favoring this frequent co-occurrence of OSAS and insomnia disorder in this specific subgroup of patients [5,45,46,47,48]. Indeed, the development of insomnia disorder in hypertensive subjects may be favored by the occurrence of anxious and/or depressive symptoms secondary to the negative psychological impact related to the presence of a chronic disease and the potential side effects of some antihypertensive medications (mainly beta-blockers) promoting the sleep fragmentation and the occurrence of nightmares following their antagonistic action on serotonergic and beta-adrenergic receptors whereas the frequent occurrence of obstructive respiratory events in hypertensive subjects may be favored by the existence of a deleterious effect of blood pressure fluctuations on upper airway muscle tone [5,45,46,47,48]. Second, high 10-year risk for CVD associated only with COMISA demonstrated in our sample of hypertensive subjects suggests that the negative synergistic effect between OSAS and insomnia disorder could have a central implication in the development of CVD for this specific subgroup of patients. However, given the data available [49,50,51], this potential central implication of the negative synergistic effect of COMISA on the 10-year risk for CVD in hypertensive subjects could be explained by the existence of a deleterious cumulative effect of the common pathophysiological mechanisms of OSAS and insomnia disorder favoring the occurrence of CVD. Indeed, both the sleep alterations associated with insomnia disorder (short sleep duration, sleep fragmentation and hyperarousal state) and OSAS (hypoxemia/reoxygenation, sleep fragmentation, intrathoracic pressure changes and hypercapnia) may induce activation of inflammatory mechanisms, dysregulation of the sympathetic nervous system and hyperactivation of the hypothalamic–pituitary–adrenal axis that play a central role in the development of CVD through changes in blood pressure/heart rate, increased atherosclerosis, occurrence of insulin resistance and increased lipid levels [52,53]. Moreover, similar to the available data [14], we have shown that this cumulative deleterious effect of COMISA on the 10-year risk for CVD in hypertensive subjects seems to be mediated by short sleep duration since unlike COMISA without short sleep duration, only COMISA with short sleep duration was associated with higher cardiovascular risk in this particular subpopulation. Thus, in hypertensive subjects, it appears essential to systematically diagnose and adequately manage COMISA to open new perspectives for CVD prevention in this specific subgroup of patients.

The demonstration of this potential central implication of the negative synergistic effect of COMISA on the 10-year risk for CVD in our sample of hypertensive subjects could open new perspectives to better understand the limited efficacy of continuous positive airway pressure therapy alone on cardiovascular prevention in this specific subgroup of patients [10,11]. Indeed, since insomnia disorder may have a direct negative effect on cardiovascular outcome and compliance with OSAS treatments [54,55], the establishment of therapeutic strategies targeting only OSAS without adequate combined treatment of comorbid insomnia disorder could promote an inadequate control of the pathophysiological mechanisms favoring the occurrence of CVD in hypertensive subjects with COMISA [49,50,51]. Nevertheless, although the development of combined treatments for insomnia disorder and OSAS may open up new therapeutic options for better cardiovascular prevention in hypertensive subjects [56], the respect of some specificities of this specific subgroup of patients will be essential during the implementation of these combined therapeutic strategies of COMISA to avoid any negative impact on the conventional management of hypertension [57]. Indeed, given the potential deleterious impact of some pharmacologic approaches for insomnia disorder on blood pressure control and the limited data concerning the potential efficacy of alternatives to continuous positive airway pressure therapy to reduce blood pressure [58,59,60], the currently recommended therapeutic approach for the first-line treatment of COMISA in hypertensive subjects appears to be the combined use of cognitive behavioral therapy for insomnia and continuous positive airway pressure therapy combined with lifestyle modifications [6,57,61]. Regarding the application sequence of this combined treatment of COMISA, there is currently no consensus due to the limited data available to favor the sequential approach (completion of cognitive behavioral therapy for insomnia followed by initiation of continuous positive airway pressure therapy) or the concomitant approach (concomitant implementation of cognitive behavioral therapy for insomnia and continuous positive airway pressure therapy) [62,63]. However, despite this lack of consensus, the results of available preliminary studies seem to suggest that both the sequential approach and the concomitant approach could be equivalent options for the combined treatment of COMISA given their similar beneficial impact on insomnia complaints and adherence to continuous positive airway pressure therapy [62,63]. Finally, independently of the potential beneficial effect for the prevention of CVD, it remains essential to effectively manage insomnia disorder and OSAS (even in case of occurrence alone) in hypertensive subjects [57] since these sleep disorders may potentially negatively impact adherence to antihypertensive treatment and life quality in this specific subgroup of patients [64,65,66,67].

### Limitations

Although the encoding of the retrospective data used in this study was performed conscientiously, these data were not collected directly from the subjects by the authors, which justifies carrying out prospective studies to confirm our findings. Moreover, since this study only included hypertensive subjects, our findings cannot be extrapolated to other cardiovascular disorders. Furthermore, although the FRS may be used to calculate the 10-year risk for CVD in hypertensive subjects, it was initially validated for the general population, which requires strict compliance with these conditions of use to allow an adequate interpretation of this cardiovascular risk score in this specific subgroup of patients. On the other hand, since the number of hypertensive subjects with some insomnia subtypes based on difficulty initiating and/or maintaining sleep was too small in some categories studied to allow adequate statistical power, we decided to focus our additional analyzes only on insomnia subtypes based on sleep duration, which could limit the interpretation of some results of this study. Finally, only hypertensive subjects who agreed to stay at the Sleep Laboratory are present in our database, which could limit the generalization of our findings to all hypertensive subjects.

## 5. Conclusions

In this study, we demonstrated that high 10-year risk for CVD is frequent in hypertensive subjects. Moreover, we demonstrated that unlike its components present separately, COMISA was associated with high 10-year risk for CVD in hypertensive subjects, which seems to indicate that the establishment of a systematic research and an adapted treatment of COMISA could open new perspectives to promote a better cardiovascular outcome in this specific subgroup of patients.

## Figures and Tables

**Table 1:** Inclusion and exclusion criteria.

Inclusion Criteria	Exclusion Criteria
Hypertension diagnostic based on the WHO diagnostic criteria: mean systolic blood pressure ≥ 140 mmHg or mean diastolic blood pressure ≥ 90 mmHg or self-reported diagnosis of clinically demonstrated hypertension or taking antihypertensive medication [19]	Age < 40 years old
Absence of uncontrolled hepatic, pancreatic, pulmonary, cardiovascular, renal, autoimmune, infectious or inflammatory pathologies	Presence of previous CVD: heart failure, cerebrovascular disease, peripheral vascular disease and coronary heart disease
Absence of current or past severe psychiatric disorders; bipolar disorder, psychotic disorder and substance absence	Presence of current or past lesions or malformations: head trauma, lesions of cerebral respiratory centers, craniofacial malformations and abnormal chest deformities
Absence of parasomnias, central disorders of hypersomnolence, OSAS already treated in the past or being treated with continuous positive airway pressure therapy or mandibular advancement device and sleep apnea syndrome with predominantly central component	Pregnancy

CVD = cardiovascular disease, OSAS = obstructive sleep apnea syndrome, WHO = World Health Organization.

## Data Availability

The data presented in this study are available on reasonable request from the corresponding author.

## Supplementary Materials

The following supporting information can be downloaded at: https://www.mdpi.com/article/10.3390/life13061379/s1, References [68,69] are cited in the supplementary materials. Annex S1: Procedure used for outpatient recruitment of hypertensive subjects; Annex S2: Diagnostic criteria used for traditional cardiovascular risk factors; Annex S3: Blood pressure measurement method; Annex S4: Description of self-questionnaires used; Annex S5: Stay conditions at the Sleep Laboratory and Applied polysomnography-montage; Annex S6: Scoring criteria used for polysomnographic recordings; Annex S7: Research Diagnostic Criteria for insomnia disorder; Annex S8: Description of the confounding factors included in the univariate analyses.

## Abbreviations

CVD, Cardiovascular Disease; DSM, Diagnostic and Statistical Manual of Mental Disorders; FRS = Framingham Risk Score; OSAS, Obstructive Sleep Apnea Syndrome.
