# Supplementary material for: 10-Year Risk for Cardiovascular Disease Associated with COMISA (Co-Morbid Insomnia and Sleep Apnea) in Hypertensive Subjects
Source: Life (Basel). 2023 Jun 13;13(6):1379. doi: 10.3390/life13061379 (PMC10303626; doi:10.3390/life13061379)
Supplement: Supplementary file 1 [file life-13-01379-s001.zip › life-2392861-supplementary.pdf]

Supplementary Data

Annex S1

Procedure used for outpatient recruitment of hypertensive subjects

All hypertensive subjects recruited for this study were initially referred to the Sleep Laboratory by physicians specialized in sleep medicine after an outpatient consultation during which a preliminary assessment of their complaints related to sleep, their ongoing psychotropic/somatic treatments and their somatic/psychiatric comorbidities was systematically performed to allow a first diagnostic hypothesis. Following this initial assessment, a polysomnographic recording was programmed in all these hypertensive subjects to allow an objective assessment of their sleep complaints and to exclude the presence of comorbid sleep disorders that could negatively impact blood pressure control.

Annex S2

Diagnostic criteria used for traditional cardiovascular risk factors

Type 2 diabetes was defined as present when one or more of the following criteria was present at admission: glycated hemoglobin (HbA1c) ≥6.5% or fasting plasma glucose ≥126mg/dl or two-hour plasma glucose ≥200 mg/dl during an oral glucose tolerance test or random plasma glucose ≥200 mg/dl in patients with classic symptoms of hyperglycemia or self-reported diagnosis of clinically demonstrated type 2 diabetes or taking antidiabetic medication [20]. In the absence of unequivocal hyperglycemia, criteria 1–3 should be confirmed by repeat testing. In addition, diabetes must have begun in adulthood [20].

Dyslipidemia was defined as present when one or more of the following criteria was present at admission: plasma triglyceride level ≥150 mg/dl or plasma HDL-cholesterol level <40 mg/dl for men or plasma HDL-cholesterol level <50 mg/dl for women or self-reported diagnosis of clinically demonstrated dyslipidemia or use of treatment for dyslipidemia [21].

Cardiovascular comorbidities were considered present when at least one of the following pathologies was present: cardiac arrhythmias, non-ischemic cardiomyopathy, cardiac valve disease and history of cardiac surgery.

Annex S3

Blood pressure measurement method

Systolic and diastolic blood pressures were manually measured at the right arm after five minutes of rest in a sitting position by well-trained nurses. For subjects with a systolic blood pressure ≥140 mmHg and/or a diastolic blood pressure ≥90 mmHg, blood pressures were again measured twice after a systematic rest period of five additional minutes. The first measurement was excluded whereas the second and third measurements were averaged to minimize the impact of white coat effect. In the absence of prior diagnosis of hypertension, pathological blood pressures were confirmed by repeated measurements during the stay at the sleep laboratory [19].

Annex S4

Description of the self-questionnaires used

- The Beck Depression Inventory (reduced to 13 items) was used to investigate the presence of depressive symptoms. The 13 items of this scale may be scored from 0 to 3, which mean that the total score may vary from 0 to 39. A final score of 0-4 indicates an absence of depressive symptoms, 5-7 mild depressive symptoms, 8-15 moderate depressive symptoms, and ≥16 severe depressive symptoms [27].
- The Epworth Sleepiness Scale was used to investigate daytime sleepiness. The 8 items of this scale assessing sleepiness in different daytime situations may be scored from 0 to 3, which mean that the total score may vary from 0 to 24. A final score greater than 10 indicates excessive daytime sleepiness [28].
- The Insomnia Severity Index was used to investigate the severity of insomnia complaints. The 7 items of this index may be scored from 0 to 4, which mean that the total score may vary from 0 to 28. A final score of 0-7 indicates an absence of insomnia complaints, 8-14 subclinical insomnia complaints, 15-21 moderate insomnia complaints, and 22-28 severe insomnia complaints [29].

Annex S5

Stay conditions at the Sleep Laboratory

The patients went to bed between 22:00 - 24:00 and got up between 6:00 - 8:00, following their usual schedule. During bedtime hours, the subjects were recumbent and the lights were turned off. Daytime naps were not permitted.

Applied polysomnography-montage

- Two electro-oculogram channels
- Three electroencephalogram channels
- One submental electromyogram channel
- Electrocardiogram
- Pressure cannula to detect the oro-nasal airflow
- Finger pulse-oximetry
- Microphone to record breathing sounds and snoring
- Plethysmographic inductive belts to measure thoracic and abdominal breathing
- Anterior tibialis electrodes

<u>Annex S6</u>

<u>Scoring criteria used for polysomnographic recordings</u>

Obstructive apneas were scored if the decrease in air flow was ≥90% for at least 10 seconds whereas obstructive hypopneas were scored if the decrease in airflow was ≥30% for at least 10 seconds with a decrease in oxygen saturation of 3% or followed by micro-arousal. The obstructive apnea-hypopnea index corresponds to the total number of obstructive apneas and hypopneas divided by the period of sleep in hours [32].

Periodic limb movements were scored on the basis of the following strict criteria: 1) duration between 0.5 to 10 seconds, 2) interval between 5 and 90 seconds from leg movement onset and 3) movements had to be part of a series of ≥4 consecutive movements meeting these criteria. Periodic limb movement index corresponds to the total number of periodic limb movements divided by period of sleep in hours [33].

Annex S7

Research Diagnostic Criteria for insomnia disorder

**Criteria**	
A	The individual reports one or more of the following sleep related complaints:<br>1. difficulty initiating sleep<br>2. difficulty maintaining sleep<br>3. waking up too early<br>4. sleep that is chronically nonrestorative or poor in quality
B	The above sleep difficulty occurs despite adequate opportunity and circumstances for sleep
C	At least one of the following forms of daytime impairment related to the nighttime sleep difficulty is reported by the individual:<br>1. fatigue/malaise<br>2. attention, concentration or memory impairment<br>3. social/vocational dysfunction or poor school performance<br>4. mood disturbance/irritability<br>5. daytime sleepiness<br>6. motivation/energy/initiative reduction<br>7. proneness for errors/accidents at work or while driving<br>8. tension headaches and/or GI symptoms in response to sleep loss<br>9. concerns or worries about sleep

Annex S8

Description of the confounding factors included in the univariate analyses

After a review of the literature on cardiovascular risk factors [14,15,68,69], potential confounders included in this study were body mass index categories (categorized: <25 kg/m^2, ≥25 & <30 kg/m^2, ≥30 kg/m^2), age (categorized: <65 years, ≥65 years), sleep movement disorders (categorized: absent, periodic limb movement syndrome alone, restless legs syndrome alone or combined with periodic limb movement syndrome), hypertension status (categorized: controlled, untreated, uncontrolled), dyslipidemia status (categorized: absent, without statin therapy, with statin therapy), CRP levels (categorized: <1 mg/L, ≥1 mg/L) and as binary variables: gender, alcohol consumption, smoking, excessive daytime sleepiness, type 2 diabetes, cardiovascular comorbidities, aspirin therapy and depression.
